# Common neurobiological correlates of resilience and personality traits within the triple resting-state brain networks assessed by 7-Tesla ultra-high field MRI

**DOI:** 10.1038/s41598-021-91056-y

**Published:** 2021-06-02

**Authors:** Dilsa Cemre Akkoc Altinok, Ravichandran Rajkumar, Dominik Nießen, Hasan Sbaihat, Margo Kersey, N. Jon Shah, Tanja Veselinović, Irene Neuner

**Affiliations:** 1 Department of Psychiatry, Psychotherapy and Psychosomatics, RWTH Aachen University, Pauwelsstraße 30, 52074 Aachen, Germany; 2 JARA – BRAIN – Translational Medicine, Pauwelsstraße 30, 52074 Aachen, Germany; 3 Institute of Neuroscience and Medicine, INM-4, Forschungszentrum Jülich GmbH, Wilhelm-Johnen-Straße, 52428 Jülich, Germany; 4 Department of Medical Imaging, Arab-American University Palestine, AAUP, Jenin, Palestine; 5 Institute of Neuroscience and Medicine 11, INM-11, JARA, Forschungszentrum Jülich, Jülich, Germany; 6 Department of Neurology, RWTH Aachen University, Pauwelsstraße 30, 52074 Aachen, Germany; 7 Department of Mathematics, University of California, Los Angeles, CA 90095 USA

**Keywords:** Translational research, Neural circuits, Human behaviour, Stress and resilience

## Abstract

Despite numerous studies investigating resilience and personality trials, a paucity of information regarding their neurobiological commonalities at the level of the large resting-state networks (rsNWs) remains. Here we address this topic using the advantages of ultra-high-field (UHF) 7T-MRI, characterized by higher signal-to-noise ratio and increased sensitivity. The association between resilience, personality traits and three fMRI measures (fractional amplitude of low-frequency fluctuations (fALFF), degree centrality (DC) and regional homogeneity (ReHo)) determined for three core rsNWs (default mode (DMN), salience (SN), and central executive network (CEN)) were examined in 32 healthy volunteers. The investigation revealed a significant role of SN in both resilience and personality traits and a tight association of the DMN with resilience. DC in CEN emerged as a significant moderator for the correlations of resilience with the personality traits of neuroticism and extraversion. Our results indicate that the common neurobiological basis of resilience and the Big Five personality traits may be reflected at the level of the core rsNWs.

## Introduction

The increasing availability of ultra-high-field systems (UHF) has opened new possibilities in many domains of neuroscience. One of the main advantages of this new technology is a more sensitive observable blood oxygenation level-dependent (BOLD) contrast change, as a result of increased susceptibility effects at UHF. This reduces nonspecific mapping signals from large vessels and accentuated microvasculature contributions and enables significantly improved functional MRI (fMRI) experiments^1^.

The most commonly used UHF systems are those operating at 7T. The clinical applicability of UHF MRI has been particularly demonstrated for the diagnosis and assessment of brain tumours, epilepsy, multiple sclerosis, cerebrovascular diseases and Alzheimer´s disease^2–6^. Considerable progress has also been made in the efforts to exploit the greater sensitivity of UHF imaging for the establishment of neuroimaging biomarkers for neuropsychiatric disorders, such as depression, post-traumatic stress and schizophrenia^7^.

Nevertheless, modern medicine is not only concerned with the treatment of diseases; it is also concerned with understanding the basics of maintaining health despite negative influences. Particularly in the field of mental health, a deeper understanding of the psychological adjustment processes is crucial in order to identify protective factors preventing mental diseases. In this context, in recent decades numerous neuroscientists have dedicated their efforts to the topic of resilience. Psychological resilience is defined as a dynamic process encompassing positive adaptation within the context of significant adversity^8^. This complex construct includes psychological (personality traits, individual resources), biological, and environmental (social and family relationships, also society and culture) factors^9^ which all influence each other in a most sophisticated way.

Thereby, particularly close association has been observed between resilience and personality traits. One of the most established models of personality structure is the Five-Factor Model (Big Five)^10^, which is commonly assessed by the NEO personality inventory (NEO)^11^. The Big Five personality dimensions or traits (i.e., neuroticism, extraversion, openness to experience, agreeableness and conscientiousness) describe enduring, cross-culturally validated, individual traits that are known to influence numerous important health outcomes and also have a strong association with resilience^12^. In adults, several investigations revealed a characteristic pattern of low neuroticism and higher extraversion, openness, agreeableness, and conscientiousness to be associated with higher resilience^13,14^.

In terms of the neurobiology of resilience, neuroimaging methods have revealed several particularities relating to structural differences in the brain and to structural and functional connectivity patterns in individuals who have been exposed to traumatic experiences without a consecutive development of trauma-related disorders^15^. A particularly prominent role has been attributed to neuronal circuitries for emotions and arousal, including multiple subcortical and cortical regions, namely the amygdala, hippocampus, insula, medial prefrontal cortex (PFC) and the anterior cingulate cortex (ACC)^16^, which are all considered as a part of the canonical resting state networks. Furthermore, a recent literature-review suggests increased default-mode-network (DMN) and salience network (SN) connectivity as a marker of higher vulnerability and lower resilience^17^.

Recent investigations indicate, that the DMN and the SN, together with the central executive network (CEN), stand out for their importance and synchronised interplay among the resting-state networks (rsNWs)^18^. These three networks are often jointly referred to as the triple network model (TNM)^19^ and are considered to be the core neurocognitive networks due to their involvement in a wide range of cognitive tasks^20^. Even more, disruptions in the coordinated activity of the TNM networks seems to play a crucial role in various psychiatric diseases^19,21^. The partially overlapping dysfunctions in these three networks has led to the hypothesis that the triple network is the final pathway where multiple etiological factors converge to produce the clinical picture in neuropsychiatric disorders^22^.

Regarding the role of the TNM in resilient individuals, the current literature mostly focuses on findings in patients with post-traumatic stress disorder (PTSD). A comprehensive review of those studies^17^ reported as a common finding in PTSD individuals reduced DMN connectivity and increased SN connectivity. There was also a significant increase in connectivity between DMN and SN^23^. In terms of functional activity, higher vulnerability was associated with increased activity in SN regions^23^ and also decreased activity with the CEN^17,24^ in this population. Furthermore, a comprehensive literature review attributes increased resting-state DMN activity and connectivity, impaired SN-mediated switching between the DMN and CEN, and ineffective CEN modulation of the DMN to an increased predisposition to major depression^25^.

The triple rsNWs have also been associated with the Big Five personality dimensions, linking the dimensions of extraversion and agreeableness to the DMN^26^, conscientiousness to higher functional connectivity in the CEN and the DMN^27^, and openness to higher connectivity of the DMN with other networks^28^. Moreover, machine-learning approaches recently showed that it might be possible to predict the Big Five personality traits from resting-state functional connectivity patterns in intrinsic brain networks^29,30^.

Despite the huge number of studies investigating resilience and personality trials, our understanding of their neurobiological commonalities at the level of the large resting-state networks is sparse. In one previous study, Killpatrick and colleagues determined a resilient personality profile by performing a factorial analysis of the NEO subscale scores and reported significant associations with the resting-state connectivity of the SN and DMN^31^. However, a direct measure of resilience using an established scale was not performed in this study.

Thus, we chose to elucidate the common neurobiological basis underlying the resilience and personality trials in healthy volunteers with regard to the triple rsNWs. The application of UHF technology enabled a highly accurate characterisation of the three networks. Here we chose three different resting-state measures, each reflecting different properties of the brain networks: the fractional amplitude of low-frequency fluctuations (fALFF)^32^, the regional homogeneity (ReHo)^33^ and the degree centrality (DC)^34^. These measures were selected as fALFF is considered an indicator of the regional intensity of spontaneous fluctuations in the blood oxygen level-dependent signal (BOLD)^32^, ReHo is commonly used to characterise spontaneous brain activity at a limited anatomical distance, and DC is applied to characterise the correlation of the BOLD signal between spatially distant brain areas at a voxel level. Thus, ReHo and DC are considered to be mutually complementary for detecting both local and remote brain activity synchronisation^35^. Together with the fALFF measure, these fMRI metrics enable comprehensive rsNW characterisation, displaying a pattern of resting-state activity (RSA), regional temporal integration and connectivity.

We hypothesised that a lower RSA and weaker regional and global connectivity in the DMN and SN would yield higher resilience scores, indicating less distraction through internal mental states and lower demand for emotional regulation in highly resilient individuals. Furthermore, we expected higher resilience to be associated with lower levels of neuroticism and higher scores on the other four NEO scales (as already established), and that these associations would be moderated by the activity and connectivity patterns of the three resting-state networks. By using the UHF technology, we expected to obtain much higher precision and accuracy, with a higher individual contribution, than the widely used fMRI technologies with lower field strength.

## Methods

### Participants

The study included 32 participants who were recruited in Aachen, Germany via online advertisements and postings. All participants were required to be healthy, with no history of neurological or psychiatric disorder, aged between 18 and 51 years, right-handed, and have no contraindications for magnetic resonance imaging (MRI). The Structured Clinical Interview for DSM-IV (SCID-I)^36^ was used to eliminate subclinical psychiatric diseases. Two short versions of the Early Trauma Inventory (ETI) were used to exclude all possible traumatic events (ETI-TL, 18 items trauma list) and trauma symptoms (ETI-TL, 23 items trauma symptoms)^37^. Written and informed consent was obtained from all participants, following the recommendations of the Declaration of Helsinki. All methods were performed according to the relevant guidelines and regulations. The Ethics Committee of the Medical Faculty of the RWTH Aachen University approved the procedures of this investigation.

### Experimental design

After inclusion in the study, participants were asked to provide demographic information and complete the two questionnaires described below. Both questionnaires do not refer to a specific time period, but they ask for a general extent of agreement with specific statements. Subsequently the MR data were acquired.

### Resilience Questionnaire (RS-25)

Psychological resilience was assessed using a German version of the 25-item Resilience Questionnaire (RS-25)^38^. The total score of the RS-25 ranges between 25 and 175^39^. The scale has a unidimensional structure and includes items such as “I am usually able to find solutions to solve problems” and “My life is meaningful.” Participants were required to rate their agreement on a 7-point scale ranging from 1-strongly disagree to 7-strongly agree. Scores exceeding 161 indicate a very high degree of resilience, scores between 146 and 160 indicate a high degree of resilience, scores between 131 and 145 indicate a moderate degree of resilience, scores between 116 and 130 indicate a low degree of resilience and scores below 100 indicate a very low degree of resilience capacity^40^.

### NEO personality inventory

The Revised NEO Personality Inventory (NEO-FFI)^41^ is a 60-item scale and provides evidence of the five different dimensions of personality: Neuroticism, Extraversion, Openness to Experience, Agreeableness and Conscientiousness. In this study, each dimension was measured with 12 defined items, and participants responded to each item using a 5-point Likert scale, with response options ranging from strongly disagree to strongly agree. Negatively worded items were reverse coded before all analyses. The NEO-FFI shows validity after being translated into German^42^.

### MR data acquisition

The MR data were acquired using the 7T MAGNETOM Terra scanner (Siemens Healthineers, Erlangen, Germany) installed at the Institute of Neuroscience and Medicine-4 (Medical Imaging Physics) (INM-4), Forschungszentrum Jülich. The structural and functional data were acquired in a single session. The scanning parameters are explained below.

#### Structural MR-imaging

Anatomical images were acquired with a T_1_ weighted MP-RAGE sequence within a scan time of 9:15 min. The matrix size was set to 256 × 256x192 to achieve a 0.8 mm isotropic resolution in the respective field of view (FOV). A short echo time (TE) 2.27 ms and long repetition time (TR) of 4500 ms was used. T_1_ weighting was introduced with an inversion time (TI) of 1000 ms. The signal to noise ratio (SNR) was optimised by using a flip angle of 4°.

#### Functional imaging

The resting-state fMRI data were acquired using spin-echo planar imaging (EPI) with an echo and repetition time, TE/TR, of 25 ms/2200 ms. A total of 273 fMRI volumes were acquired within a 10.05 min acquisition time, with 36 slices at a slice thickness of 3.1 mm. The image matrix size was 64 × 64 and the FOV was 200 × 200 mm^2^, resulting in a 3.1 mm isotropic resolution. Subjects were instructed to lie in the scanner with their eyes closed and think of nothing specific.

### RS-fMRI data processing

The RS-fMRI data were analysed using MATLAB based software packages (The Math Works, Inc., Natick, MA, USA) such as data processing and analysis for brain imaging (DPABI) (Yan et al., 2016), and SPM12 (http://www.fil.ion.ucl.ac.uk/spm/). The RS-fMRI data pre-processing was conducted by firstly removing 10 image volumes^43^, followed by slice timing correction, realignment, nuisance covariates regression (NCR) and temporal filtering between 0.01 and 0.1 Hz. The NCR covariant involved Friston’s 24 parameter modal for head motion^44^ and for the mean signals from the whole brain white matter and cerebral spinal fluid. The fMRI measures were calculated for each subject separately in individual brain image space. The DC was computed by using a Pearson correlation coefficient between the time series of a given voxel and all other voxels in the whole brain by thresholding each correlation at (r > 0.25, p ≤ 0.001)^45^. ReHo was calculated by estimating the similarity between the time series of a given voxel and its 26 neighbouring voxels using Kendall’s coefficient of concordance (KCC). The usage of 27 voxels is considered as appropriate for covering all directions in 3D space and optimizing the trade-off between mitigation of partial volume effects and generation of Gaussian random fields^46^.

The fALFF was calculated within the low-frequency range (0.01–0.1 Hz)^32^. The fMRI measures were linearly standardised into Z-values by subtracting the mean whole-brain voxel value from each voxel and then dividing the difference by the standard deviation of the whole brain. The Z-value standardised fMRI measures were spatially normalised to the MNI152 standard space (2 mm × 2 mm × 2 mm) using DARTEL algorithm^47^. Finally, spatial smoothing was performed using the Gaussian Kernel size of 4 mm^3^.

### Triple rsNW definition

Masks of the triple rsNWs were obtained from 90 functional regions of interest (fROIs)^48^. In order to further restrict the analysis to the grey matter (GM) regions, only the voxels within the GM region of the RSNs (which show more than 50% probability of being GM) were considered (GM correction). A GM tissue-segmented MNI152 (2 × 2 × 2 mm^3^) template (GM mask) was used for GM correction. A whole-brain GM mask was created by only considering the voxels that showed more than 50% probability of being GM from the tissue-segmented MNI152 template. Finally, all voxel values within the GM-corrected triple RSNs were extracted from the previously calculated fMRI measures.

### Statistical analysis

Statistical analyses were performed using the MATLAB (The MathWorks, Inc., Natick, United States) and SPSS software packages (IBM Corp. Released 2017. IBM SPSS Statistics for Windows, Version 25.0. Armonk, NY: IBM Corp.). The mean values of the RS-fMRI measures were calculated for each subject within the considered regions (DMN, SN, and CEN). In order to determine the association between the neuropsychological score (RS-25, NEO-FFI) and mean RS-fMRI measures, two-tailed Pearson linear correlation coefficients (r) were computed with a significance level of 5%. The family-wise error rate (FWER), due to multiple comparisons in the Pearson correlation analyses, was controlled for using a permutation test^49^. For each comparison (correlation), 10^5^ permutations were performed, and the p-value was adjusted using the “max statistics” method^49^.

**Figure 1:**
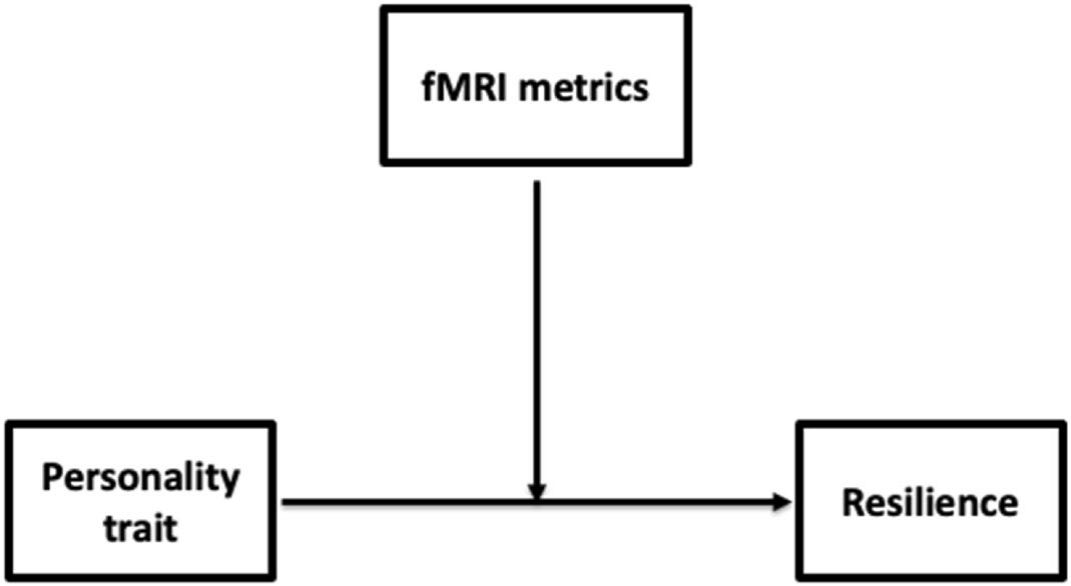
Conceptual model for the simple moderation analysis. Only personality trials, that were significantly associated with the resilience (extraversion, conscientiousness, neuroticism) were included in the model. The three fMRI metrics (fALFF, ReHo, DC) were included in the model as moderators. Age was considerate as a covariate.

A moderation analysis was performed to determine whether the relationship between resilience and personality traits depends on (or is moderated by) the fMRI metrics (fALFF, ReHo, DC). Moderation analysis is used when one is interested in testing whether the magnitude of a variable’s effect on some outcome variable of interest depends on a third variable or set of variables^50^. Here, the SPSS Macro Process^51^ was used. For the simple moderation analysis, the model 1 (linear moderation) was applied, as conceptually depicted in Fig. 1. In the model the examined variables are typically set to represent low, moderate, and high values, such as a standard deviation below the mean, the mean, and a standard deviation above the mean, respectively.

## Results

### Subject characteristics

In total, 35 participants were included in the study. Two left-handed participants were excluded from the analysis. One participant was excluded due to possible PTSD Symptoms in the interpretation of the ETI-TL. Thus, the final data analysis was composed of 32 participants (16 females; mean age 28.84, SD 9.16). All of the participants were healthy according to the Structured Clinical Interview for DSM-IV (SCID-I).

### Resilience and personality characteristics

The results of the behavioural tests are presented in **Table ****1**.

**Table 1:** Mean scores achieved on the resilience Scale (RS-25) and the NEO-FFI.

	Mean	SD	Minimum	Maximum
Resilience scale (RS-25)	145.1	14.6	120	171
Neuroticism (NEO)	14.5	6.9	5	31
Extraversion (NEO)	29.2	6.5	13	42
Openness to experience (NEO)	30.8	5.4	15	42
Agreeableness (NEO)	34.4	5.2	18	44
Conscientiousness (NEO)	35.4	7.1	19	48

All individual values are given in supplementary table 1.

On the RS-25, the participants reached mean scores of 145.1 (SD: 14.6), which corresponds to a moderate to high degree of resilience^40^.

**Figure 2:**
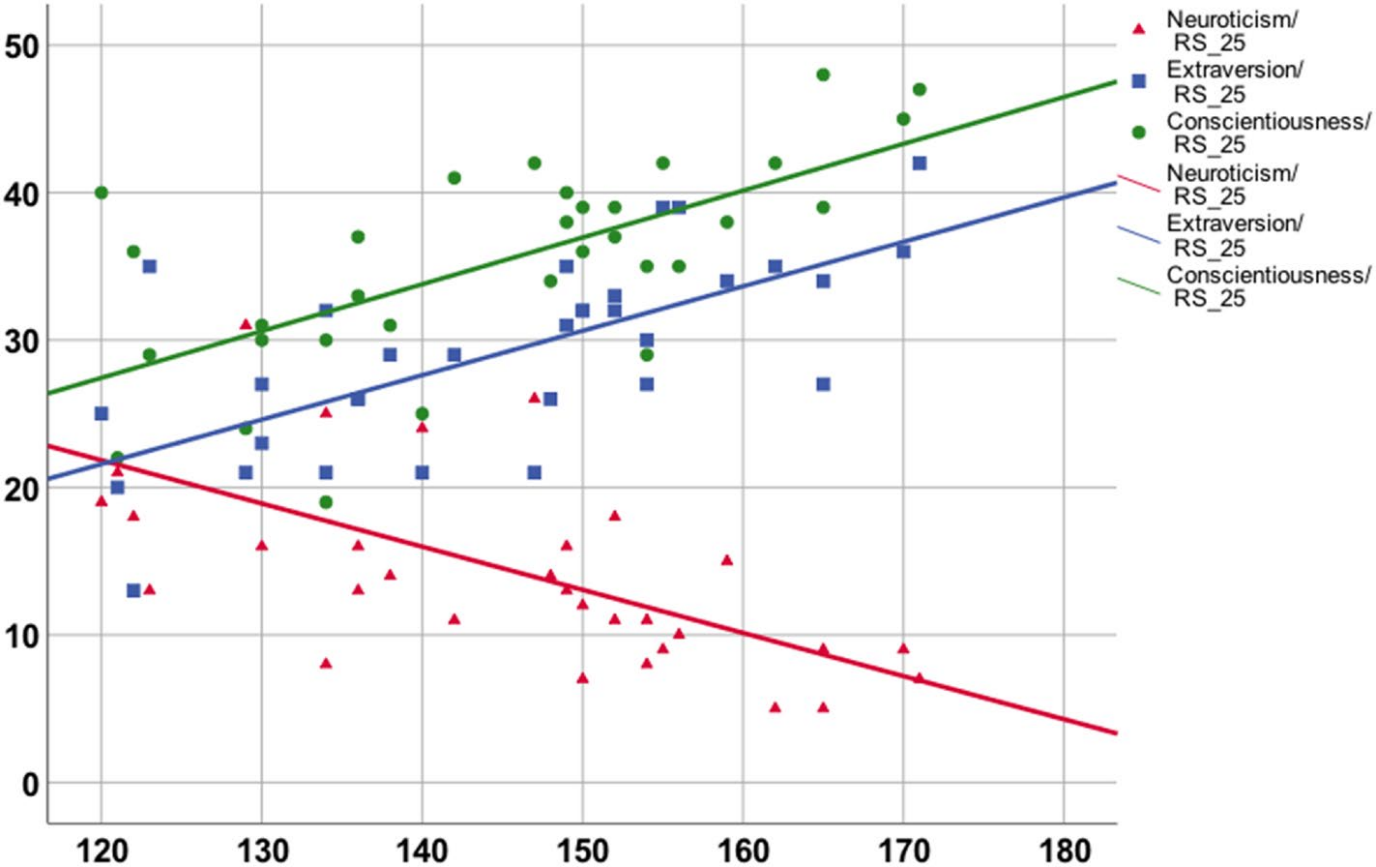
Correlations between RS-25 and three subscales of the NEO-personality scale: extraversion, conscientiousness and neuroticism.

The RS-25 showed a highly significant positive correlation with the traits’ extraversion (r = 0.674, p < 0.001) and conscientiousness (r = 0.66, p < 0.001) in the NEO-personality scale, as well as a highly significant negative correlation with neuroticism (r = -0.62, p < 0.001). These correlations (Fig. 2) remained significant after Bonferroni correction for multiple testing. The correlations with the two other personality dimensions (openness to experience and agreeableness) were not significant (p > 0.05 for both).

### RS-fMRI measures—triple network identification

**Figure 3:**
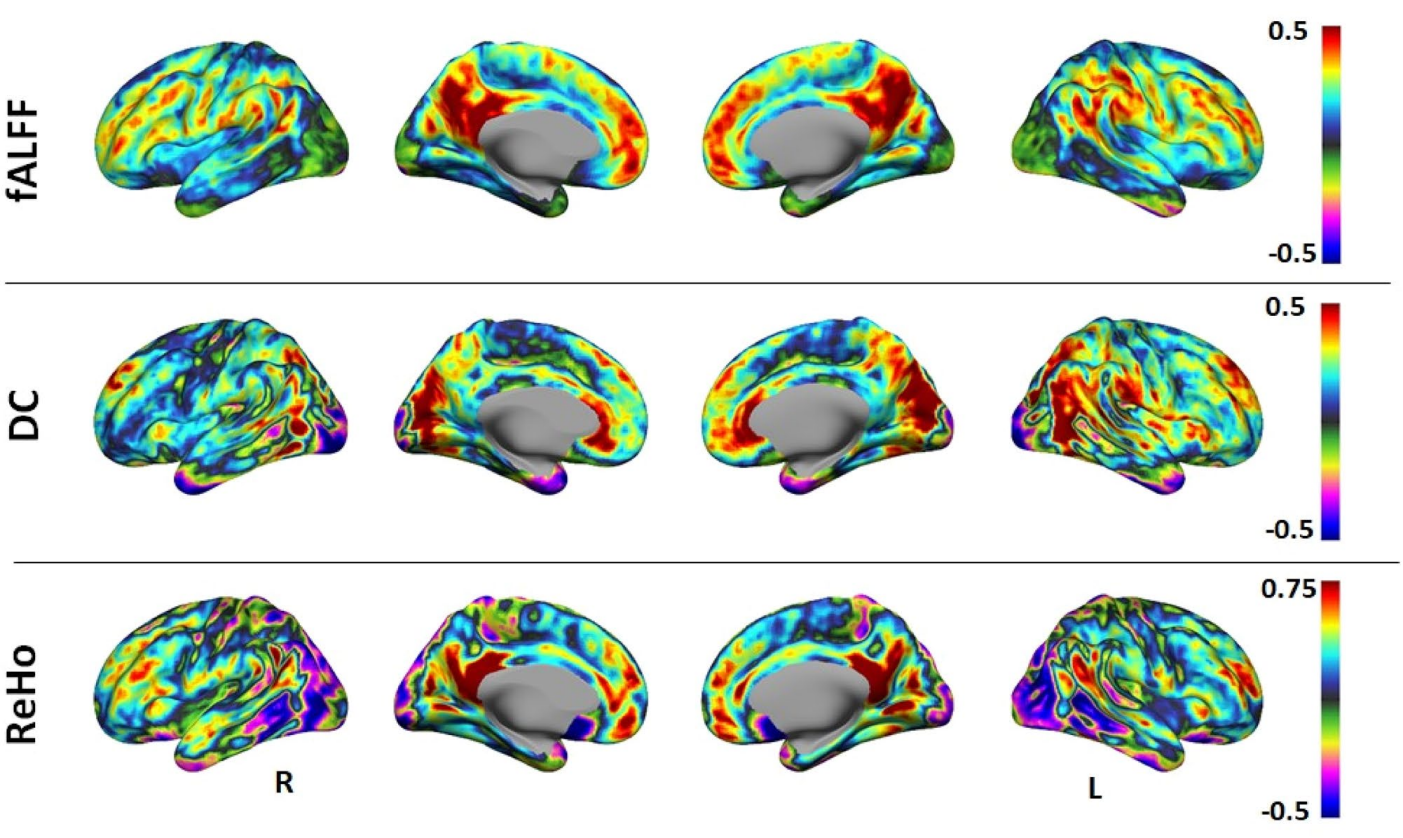
RS-fMRI measures. The fMRI measures fALFF (top row), DC (middle row) and ReHo (bottom row) are shown in right, right medial, left medial and left views. The figure was created using MATLAB (The MathWorks, Inc., Natick, United States, Version 9.5 (R2018b)) based software package CONN (version v.18.b; https://www.nitrc.org/frs/?group_id=279).

The fMRI measures were calculated as described, and the fALFF, ReHo and DC measures are depicted in Fig. 3.

**Figure 4:**
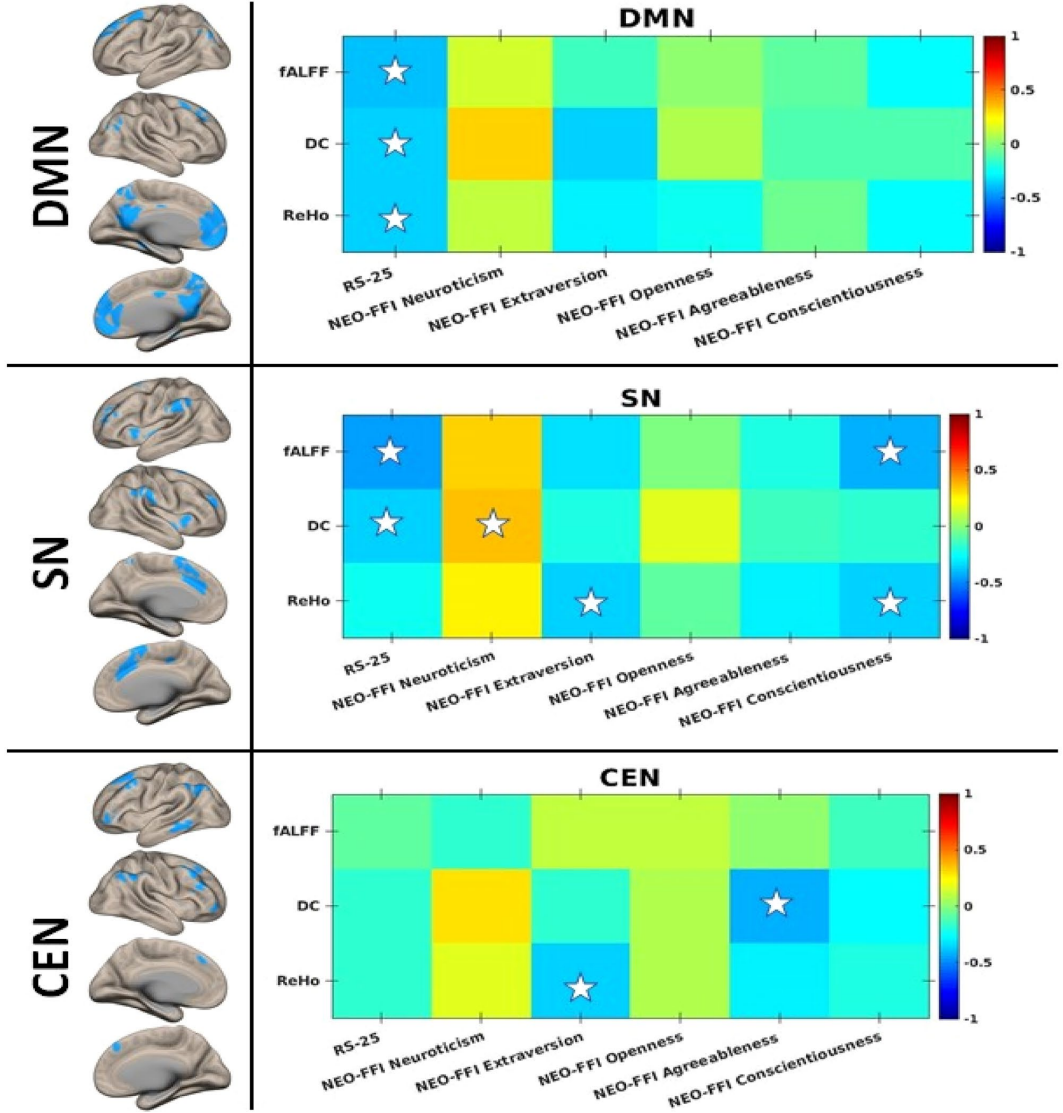
Correlation matrix head map and the depiction of the triple resting-state networks. The correlation matrix shows the Pearson correlation values between RS-25 and NEO-FFI scores with fMRI measures within the triple resting-state networks: DMN (top row), SN (middle row), and CEN (bottom row). The corresponding masks of the triple resting-state networks are shown in the right column. The colour bar indicates the Pearson correlation values from − 1 to + 1. Asterisks indicate significant correlations at the 0.05 significance level. The figure was created using MATLAB (The MathWorks, Inc., Natick, United States, Version 9.5 (R2018b) https://de.mathworks.com) and CONN (version v.18.b https://www.nitrc.org/frs/?group_id=279) software package.

The location of the triple network (Fig. 4) was obtained as described above. Specifically, the DMN included the posterior cingulate cortex (PCC), precuneus, angular gyrus, and medial prefrontal cortex (mPFC); the SN included the frontal insular cortex (FIC) and anterior cingulate cortex (ACC); the CEN included the lateral posterior parietal cortex (LPPC) and dorsolateral prefrontal cortex (DLPFC).

### Association between the fMRI metrics across the TNM networks and the behavioural measures

The correlation matrix between the fMRI metrics across the TNM networks and the behavioural measures is shown in Fig. 4.

Within the DMN, significant negative correlations between resilience scores and all three fMRI measures, fALFF (r =  − 0.39, p = 0.03), DC (r =  − 0.36, p = 0.04) and ReHo (r =  − 0.37, p = 0.03) were observed.

Within the SN, the resilience scale showed a significant negative correlation with fALFF (r =  − 0.45, p < 0.01) and DC (r =  − 0.36, p = 0.04). Significant correlations in this network were also observed for the personality tests: for extraversion with ReHo (r =  − 0.37, p = 0.03), and for conscientiousness with ReHo (r =  − 0.36, p = 0.04) and fALFF (r =  − 0.42, p = 0.01), as well as between neuroticism and DC (r = 0.037, p = 0.04).

Within the CEN, extraversion correlated significantly with ReHo (r =  − 0.37, p = 0.04). Furthermore, there was a significant negative correlation between agreeableness and DC (r =  − 0.44, p = 0.01).

**Figure 5:**
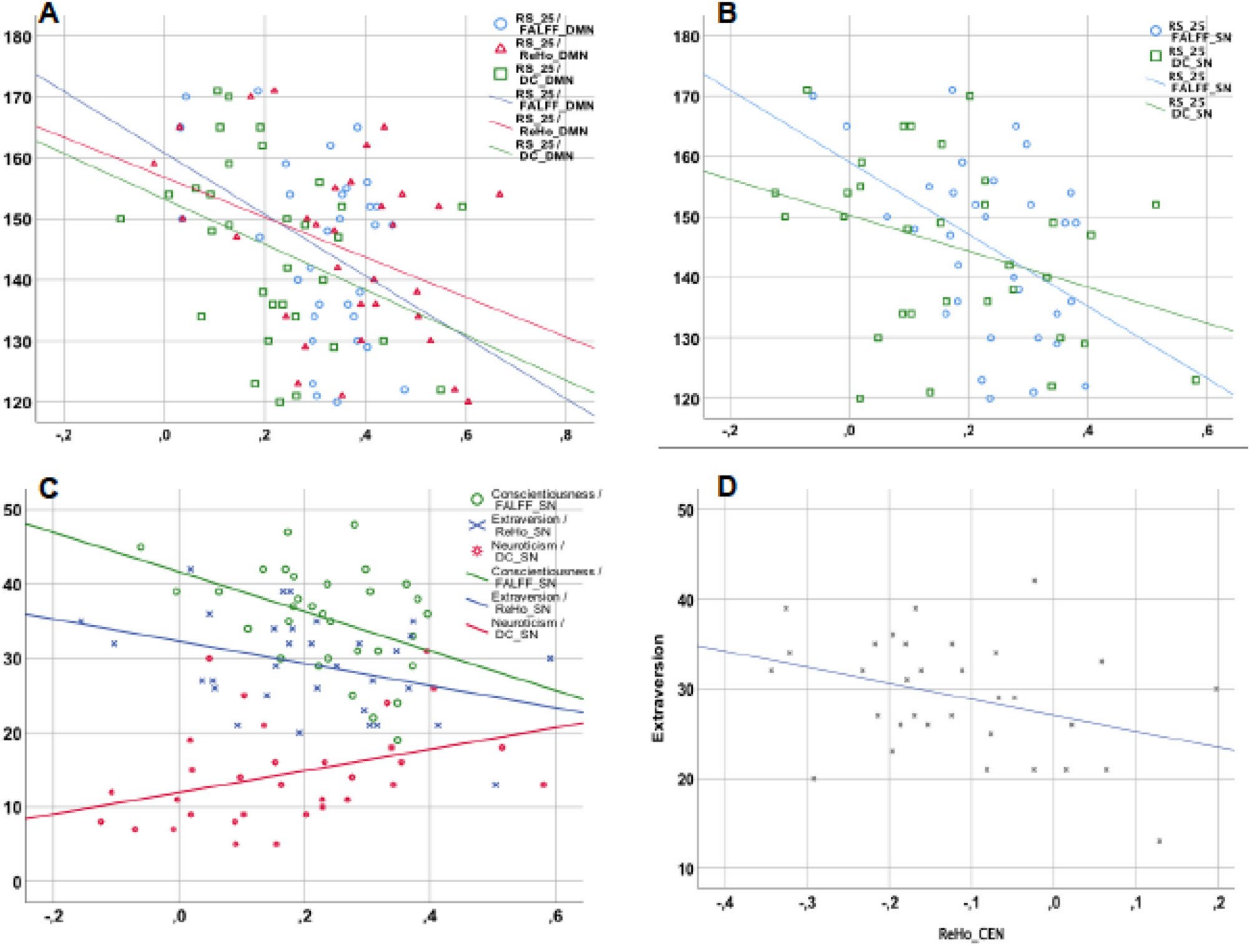
(**A**) Correlations between RS-25 and the fMRI metrics in the DMN. (**B**) Correlations between the RS-25 and the fMRI metrics in the SN. (**C**) Correlations between three personality traits (extraversion, conscientiousness and neuroticism) and the fMRI metrics in the SN. (**D**) Correlation between extraversion and ReHo in CEN.

For the purpose of better presentation, the described correlations are depicted in Fig. 5.

**Figure 6:**
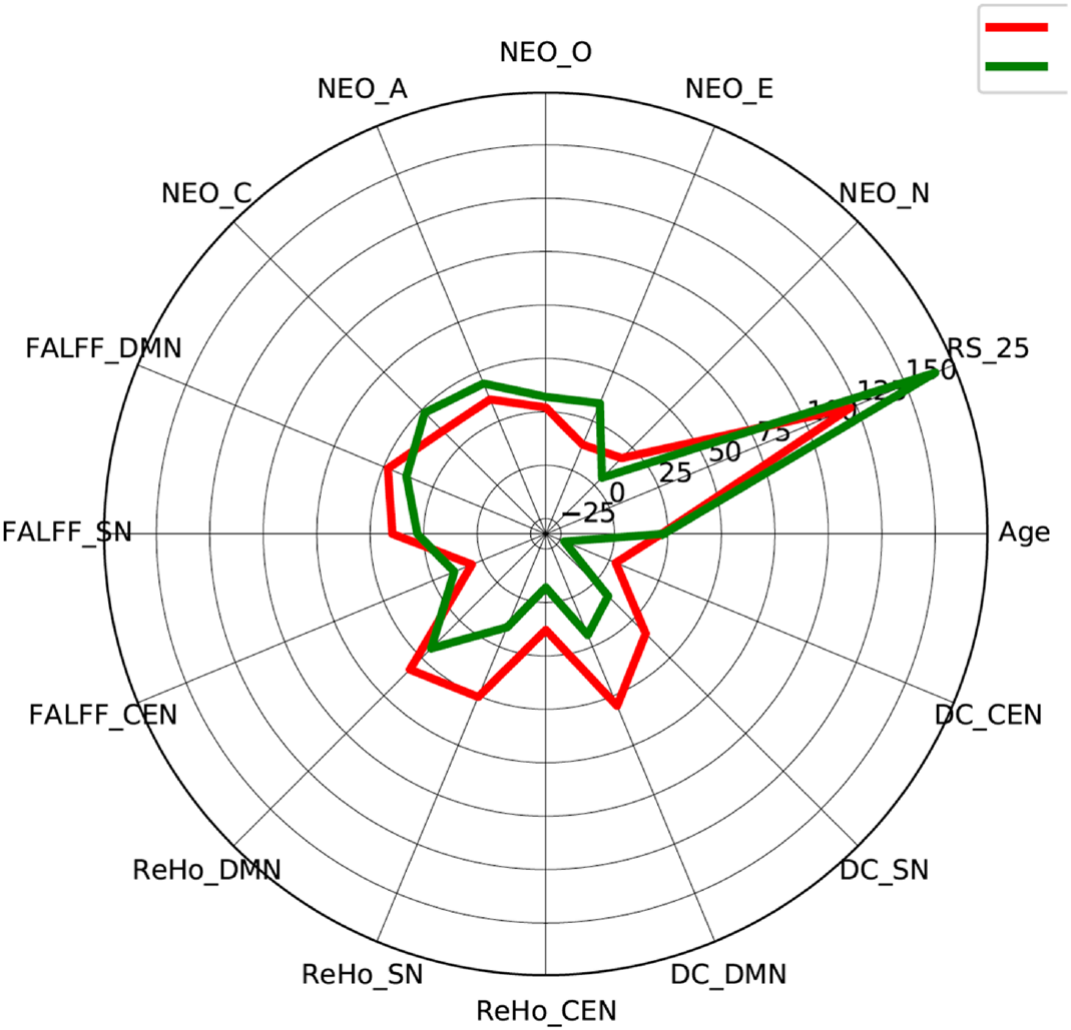
Individual depiction of the investigated behavioural (RS-25 and the 5 personality straits) and the fMRI metrics (ALFF, ReHo and DC) in the three core resting state networks for two age matched subjects: red line: male, intermediate resilience (RS = 122), 22 years and green line: male, high resilience (RS-25 = 165), 23 years. The figure was created using the Python (version 3.8.9) libraries numpy and matplotlib.

For a for an exemplary individual depiction of the investigated behavioural (RS-25 and the 5 personality traits) and the fMRI metrics (ALFF, ReHo and DC) in the three core resting state networks we show these parameters for two age matched persons in Fig. 6.

### Moderation effects of fMRI metrics on the correlation between RS-25 and the Big Five personality traits

The fMRI metrics obtained from the three resting-state core networks were examined as moderators of the relation between the RS-25 and the significantly correlating personality traits. Age was considerate a covariate.

**Figure 7:**
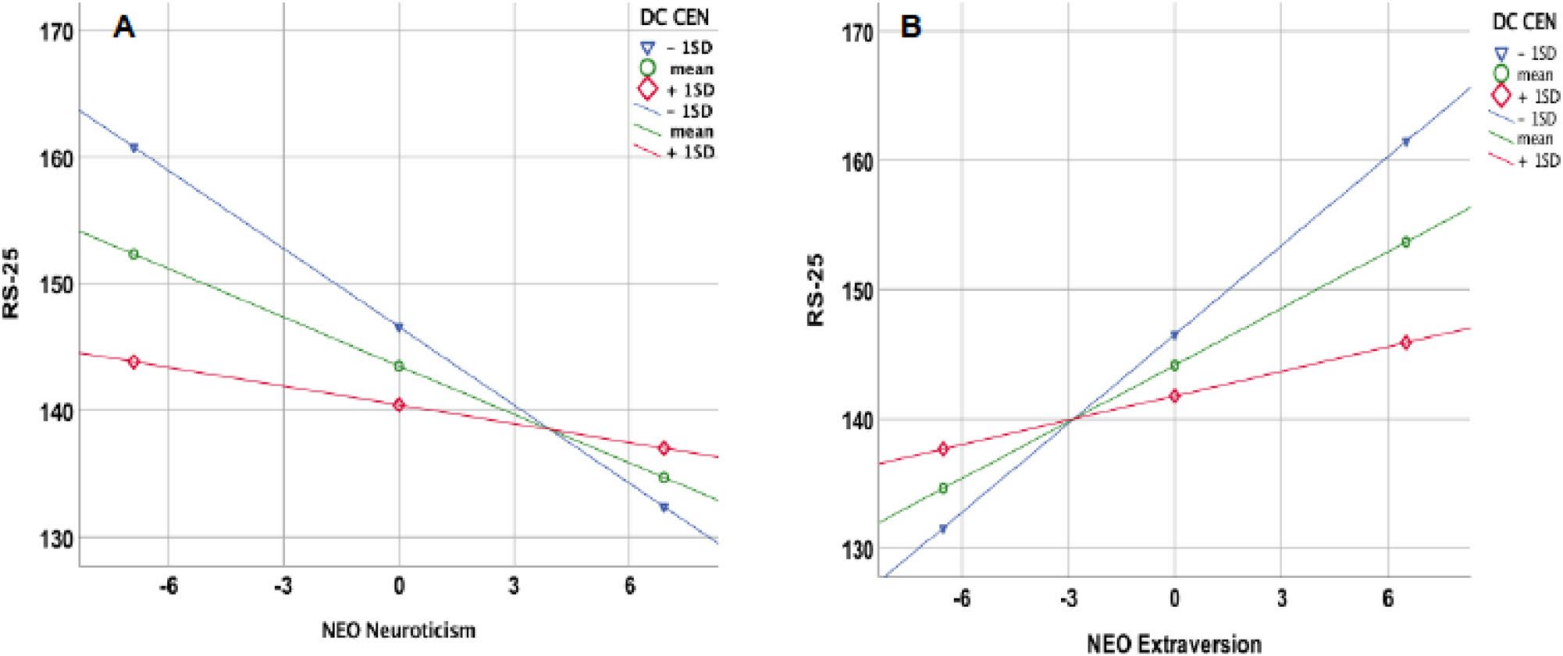
Significant moderation effect of DC in CEN obtained for the correlation of RS-25 with NEO neuroticism (**A**) and NEO extraversion (**B**).

The analysis revealed the DC in the CEN as a significant moderator of the correlation between RS-25 and neuroticism (p = 0.0052, t = 3.043). A similar moderation effect could also be observed for the DC in the CEN, showing a significant moderation effect on the correlation between R-25 and extraversion (p = 0.0099, t = 3.085) (Fig. 7).

The data that support the findings of this study are available from the corresponding author upon reasonable request.

## Discussion

In our study, ultra-high field 7-Tesla functional MRI was used to investigate the common neurobiological basis underlying psychological resilience and the Big Five personality dimensions with regard to the triple rsNWs. Our investigation revealed that the SN appears to play an important role in both resilience and personality traits. The fMRI properties of the whole DMN were found to be tightly associated with resilience but not with the personality traits. In the CEN, a significant negative correlation was only observed between the short distance connectivity parameter ReHo and extraversion. However, the DC parameter in CEN emerged as a significant moderator of the correlations of resilience with the personality traits of neuroticism and extraversion. Thereby, these correlations lost their significance in individuals with higher DC values in CEN.

Compared to earlier investigations of the resting state correlates of resilience and personality traits, the main advantage of our study was the utilisation of 7T technology. One of the principal benefits of increased magnetic field strength includes field-dependent linear gains in the signal-to-noise ratio (SNR)^52–54^ which allows better visualization of smaller structures^4,55^. Indeed, Morris and colleagues demonstrated in their recent work up to 300% improvement in temporal SNR and rsFC coefficients provided by UHF 7T fMRI when compared to 3T, indicating enhanced power for detection of functional neural architecture^56^. The UHF technology improves the confound introduced by partial volume effects^57^ with and supralinear gains in the functional contrast-to-noise ratio (fCNR)^54^ resulting in increased sensitivity to susceptibility changes^6^. Altogether, 7T can increase the conspicuity between different brain regions^6^ .These effects improve the segregation of signals originating from different anatomical substrates (e.g. microvasculature, large surface vessels, white matter), thereby enabling a significant increase in the spatial specificity. Previous investigations have shown that increased fCNR at 7T allows the comprehensive study of the distribution of spontaneous BOLD fluctuations within functionally connected networks, even at a single-subject level, without the need for extensive spatial smoothing^58^. Generally, the enormous increase in resolution in the context of UHF imaging is expected to allow for the translation of this technology into the clinic for imaging pathology at microscale level^5^ .

Another particularity of our study is the analysis according a whole network-based approach. We examined the RSA and FC within the three rsNWs that provide the basis of the TNM. Whereas previously, the properties of individual brain subregions were investigated in order to draw conclusions about the networks as a whole, we chose a network-based approach, calculating the RSA and FC parameters for the predefined networks obtained from the 90 functional regions of interest (fROIs) atlas^48^. This allows a statement to be made about the overall function of the networks, rather than the function of single subregions, which, in most cases, cannot be exclusively assigned to one network.

Consistent with previous findings, our study revealed a tight association between the resilience and personality traits captured with the NEO-questionnaire. Thereby, high resilience was associated with higher levels of extraversion and conscientiousness, as well as with lower neuroticism scores. This finding are in line with several previous reports^59^. Moreover, it has been shown, that this kind of personality profile is associated with lower tendency to develop psychopathology^60^ as well as with best subjective health in both a cross-sectional assessment^61^ and in a longitudinal observation over an 8-year period^62^. Extraversion is linked to pronounced engagement with the external world and the tendency to experience positive emotions^63^, whereas conscientiousness is characterised by self-discipline, orderliness, organisation, responsibility, and good impulse control^64^. The personality trait neuroticism is indicated by interpreting stressful conditions prominently as threatening and experiencing frequent, intense negative emotions^65^. Summing up, high extraversion and conscientiousness combined with low neuroticism appear to be the core elements of the resilient personality profile, resulting in a higher level of self-control and motivation, a higher level of positive emotions and engagement with social activity, and a higher level of emotional stability and lower level of negative emotions^66^. Our results confirm that even in a group consisting of healthy subjects with a moderate to high degree of resilience, this kind of personality constellation represents a profile which is better able to overcome stressful life events.

Following our objective to investigate the common neurobiological basis underlying the psychological resilience and the Big Five personality dimensions with regard to the triple resting-state networks, the SN was identified as playing an important role in both domains. Very concretely, the resilience score showed a significant negative correlation with spontaneous activity (fALFF) and global functional connectivity (DC) in the SN. This finding indicates that high-resilience might be associated with less switching of the SN between systems involved in processing exogenous and self-relevant information, and thus with sufficient emotional response without the interference of internal or external inputs and focus on the actual state of mind. This is in accordance with previous findings of a less prolonged response in the insula, which is the central hub of the SN, in participants with high resilience^67^. In contrast, increased intrinsic functional connectivity of the SN has been reported in borderline personality disorder, which is mainly characterised by the instability of emotion regulation^68^. Also, a hyper-activated and hyper-connected SN causing inefficient DMN-CEN modulation in PTSD patients represents a reverse construct of resilience^69^. Thus, lower spontaneous brain activity and weaker global functional connectivity in the SN in high resilient individuals may contribute to the successful filter function of the SN to balance and evaluate the internal state and external stimuli, and stand for a situation-dependent appropriate adjustment of the emotional resources.

Parallel to the described correlations with resilience, lower levels of spontaneous brain activity (represented by fALFF), regional homogeneity (ReHo) and long-range functional connectivity (represented by DC) within the SN were found to be associated with higher levels of conscientiousness, higher levels of extraversion and lower levels of neuroticism.

With respect to conscientiousness, this personality trait has traditionally been considered to be a protective factor, providing longevity and subjective well-being^70^. However, several investigations have produced evidence suggesting that conscientiousness is not always advantageous for well-being and could also be a factor that promotes stress and discomfort under adverse circumstances^71^. Indeed, Dham and colleagues demonstrated higher activation in some regions belonging to the SN (right amygdala and left insula) during an fMRI stress task conducted on highly conscientious males, indicating a disadvantageous response to uncontrollable stress^72^. In a comprehensive review^73^, the authors identified a brain network resembling a combination of the salience and ventral attention networks as a neural correlate of conscientiousness. Thereby, higher synchrony within the components was significantly positively associated with conscientiousness. Similarly, Rueter and colleagues^74^ reported a positive correlation between conscientiousness and connectivity within a network consisting of ACC, anterior insula, and middle and superior frontal gyri. In contrast to this studies, which were focused on the examination of connectivity measures within networks, we addressed other fMRI measures in our study, and found higher conscientiousness to be related to lower levels of BOLD fluctuations (fALFF) and local synchronisation (ReHo) in the SN. Our results cannot be directly compared with the findings stated above since we used different fMRI measures and addressed the whole SN, rather than parts of the network. Our finding of lower spontaneous activity and local synchronisation in the SN, as measured with a 7T fMRI, in more conscientiousness subjects, may indicate reduced distractibility and threat monitoring in the absence of relevant stimuli among these subjects. This may be a basis for a more successful prioritisation and a greater ability to focus on incoming tasks.

Regarding extraversion, a comprehensive literature review^73^ emphasises its association with function and structure in regions of the brain that are part of the reward system, indicating sensitivity to reward as a core function underlying extraversion. Here again, several resting-state investigations report a preferential association between the rewarded task responses and the SN^75^. A positive correlation between extraversion and the connectivity strength in the bilateral insula and anterior cingulate cortex (ACC) in the SN was identified using a data-driven approach^76^. Moreover, Kilpatrick and colleagues found stronger connectivity in the right anterior insula within the SN in individuals with a resilient personality profile^31^. In our study, we found a negative association between extraversion and ReHo in the SN. Keeping in mind, that ReHo is proposed as a voxel-wise synchronisation measure of the time courses of neighbouring voxels^33^ and thus reflects the degree of segregation, as opposed to the long-range connectivity measures, which reflect the degree of integration^77,78^, our results appear to be congruent with this previous reports. Consequently, the observed negative correlation between ReHo in SN and extraversion may indicate that a higher level of functional segregation in the SN may be associated with lower extraversion and thus lower reward sensitivity in healthy subjects. Also, considering the involvement of the insula in various neuropsychiatric diseases and its crucial role for emotional processing^79^, our findings link the differences in affective processing, that can be observed in individuals with high and low levels of extraversion, to the SN.

Since our results concerning the association between conscientiousness with fALFF and ReHo as well as between extraversion and ReHo measured in the whole SN were negative, while some other authors reported positive correlations of those traits with some other connectivity measures in some subregions of the SN, we performed an additional correlation analysis between this personality traits and fALFF and ReHo in the subregions of the SN, using the same methodology described in the methods part. The masks of the subregions were also obtained from an atlas of 90 functional regions of interest (fROIs). The results are given in the supplementary tables 2 and 3.

Those results obtained from the subregions of the SN will not be discussed in more detail as this does not correspond to the network-based approach defined at the beginning. However, the detection of significant correlations of conscientiousness and extraversion with the cerebellar lobule VI, deserves a brief elaboration as, to the best of our knowledge, this has not been described so far. It is already known, that cerebellum VI is involved not only in motor but also in nonmotor processes (attentional/executive and default-mode)^80^, including social behaviour and emotional processing^81^. An affiliation of this region to the salience network has been demonstrated previously^82^, indicating that it may contribute to estimating the valence of salient emotional cues and in selecting appropriate behavioural responses^82^. Our results may indicate an association of better expression of those skills with higher levels of conscientiousness and extraversion. However, due to the higher sensitivity and the significantly higher signal strength of the UHF in our study, values of the investigated parameters from all predefined subregions of the SN have been integrated into the main analysis, thus enabling statements at the network level. Such an approach would not be possible with the same reliability at lower field strengths, since the threshold for detecting the signals is much higher there.

In contrast to the negative correlations of the two fMRI metrics in the SN with extraversion and conscientiousness, the trait of neuroticism showed a positive correlation with the DC measure in this network. Several earlier studies indicate that neuroticism might have neurobiological correlates to brain regions associated with emotion processing, such as the amygdala, ACC and insula, which are also the main components of the SN^83^. In our study, a higher level of neuroticism was associated with a significantly higher level of global FC (DC). DC is a graph-based measurement of network organisation that reflects the number of instantaneous functional connections between a region and the rest of the brain, within the entire connectivity matrix of the brain (connectome)^45^. Thus, it shows how many of the nodes influence the entire brain area and its involvement in integrating information across functionally segregated brain regions^84^. In subjects with higher neuroticism, high levels of DC in the SN may indicate a higher involvement of this network in information exchange and the continuous effort of coordinating the activity of other networks^85^ across the connectome during the rest, despite the absence of truly salient information. This may be a correlate of the dysfunctional filtering of relevant stimuli and may contribute hypervigilance and hyper-arousal in individuals with high neuroticism scores. Indeed, previous investigations linked increased activity in SN subregions to hyper-arousal and hypervigilance in highly neurotic individuals^86^, which may convey a more sensitive avoidance system^87^.

Highly resilient individuals showed lower spontaneous activity (fALFF) as well as lower local and global functional connectivity (ReHo and DC) in the DMN. This result might indicate that highly resilient individuals focus less on their internal mental state, but more on the present moment, without a high input from self-referential thoughts or without creating alternative scenarios to the present, which are considered central functions of DMN^88^. Similar to our results, increased experience in mindfulness training, which is known for non-judgmental awareness of experiences in the present moment, was related to weaker functional connectivity between regions in DMN involved in self-referential processing and emotional appraisal^89^. Conversely, the hyperactivity in DMN leads to increased ruminative and obsessive thinking^90^, whereas higher resilience has been found to be associated with lower susceptibility to rumination^91^. In contrast, a tendency towards increased rumination has been indicated in many psychiatric conditions, particularly with major depression^92^, but also with anxiety^93^, PTSD^94^, and schizophrenia^95^. A meta-analysis of depression reported hyper-connectivity within the DMN^96^, which in turn seems to be associated with rumination^97^. Accordingly, the reduced suppression of the DMN during task performance in depressive patients is referred to as rumination and is associated with an abnormal increase in self-focus rather than task behaviour^98^. Aside from mental illnesses, several investigations have confirmed a link between higher resilience and happiness, defined as a composition of life satisfaction, coping resources and positive emotions, which predicts desirable life outcomes in many domains^99^. Furthermore, happiness has been related to reduced mind-wandering^100^, which is one of the critical roles of the DMN regions^101^. Correspondingly, unhappy people showed increased regional functional connectivity in the DMN^102^, whereas more successful coping styles could be related to reduced interaction between the DMN and SN nodes^103^. Taken together, lower DMN spontaneous activity and weaker connectivity, indicating less information exchange and “mind-wandering” at rest, may be a neural marker of higher resilience and indicative of a generally higher level of mental well-being.

For the third canonical resting-state core network, the CEN, we observed a significant negative correlation between the personality trait extraversion and the local functional connectivity (ReHo). Previous investigations have hypothesised that higher ReHo values indicate higher within-region synchronous activity and possibly decreased communication with remote brain regions. Thus, regions with higher ReHo are often functionally segregated from distant hubs and may compensate for the sparsity of information transfer by synchronising their activity^104^. With respect to this, our results may link decreased communication of the CEN with remote brain regions and lower levels of extraversion, in a similar way as discussed above for the SN.

Our final analysis in this study aimed to investigate the extent to which the different properties of the three networks have a moderating effect on the association between the personality traits and resilience. Here, DC in the CEN appeared to be a significant moderator of the correlation between RS-25 and neuroticism as well as for the correlation between R-25 and extraversion. In detail, both correlations only remained significant in subjects with lower DC values, whereas the correlations disappeared in subjects with greater than average DC. Our results imply that higher levels of DC, indicating a more intensive interaction with distant brain regions, enables greater flexibility and a kind of decoupling between the permanent personality traits and resilience.

Several limitations must be taken into account when interpreting our results.

One of the first concerns might arise due to the relatively small number of test persons. Had the study been a purely behavioural investigation, this would represent a major drawback. However, as the main aim of this study was to elucidate the neurobiological commonalities of resilience and personality traits using an UHF 7T fMRI, it is less of an issue. As stated above, the UHF technology offers higher SNR and fCNR, thus enabling the study of the distribution of spontaneous BOLD fluctuations within functionally connected networks in single subjects^58^. Therefore, our sample appears representative, especially since on behavioural level the associations between resilience and personality traits replicate the findings from previous reports in larger samples.

Further, previous investigations indicate sex differences in the relationship between psychological resilience and regional resting-state brain activity^105^. Due to the sample size, we abstained from comparing female and male participants. To extend our findings, future studies with a larger sample size should include a diverse age range and specifically examine the female and male intrinsic brain network activations and connections.

Furthermore, the question of the longitudinal stability of the associations reported here is of particular interest. Especially considering that resilience is a dynamic construct, future studies should address the question of the extent to which longitudinal changes in the resting state allow conclusions to be drawn about changes in individual psychological resilience.

## Conclusion

Our study represents, to the best of our knowledge, the first investigation using UHF 7-Tesla functional MRI, to determine the common neurobiological basis underlying psychological resilience and the Big Five personality dimensions in regard to the TNM. Using a whole-network approach, the investigation revealed a significant role of the SN for both, resilience and personality traits, indicating the importance of proper stimulus filtering for healthy emotion regulation and better attention control, promoting resilient personality. Consequently, stronger global connections of the SN may imply a particular tendency to neuroticism. Furthermore, a lower spontaneous activity, as well as lower local and global functional connectivity (ReHo and DC) in the DMN, also emerged as properties of high resilience.

By taking advantage of UHF imaging, our study was able to capture a subtle granularity of the examined fMRI parameters, despite the fact that our sample consisted of a relatively homogeneous group of healthy participants with moderate to high resilience. This was feasible due to the significantly higher signal strength that could be achieved in the UHF. In fact, previous findings on the neurobiological correlates of resilience are mainly based on studies in which resilient individuals (with previous traumatic experiences) or patients with a trauma sequel disorders were compared with healthy individuals^17^. The high signal strength in the UHF approach enabled us to detect subtle differences in the healthy subjects in our study, without a need for a contrast to other groups with mental pathology.

To conclude, our results indicate that the common neurobiological basis of resilience and the Big Five personality traits may be reflected in the core rsNWs.

## Supplementary Information

Supplementary Table 1.

Supplementary Table 2.

Supplementary Table 3.
