# Supplementary material for: Common neurobiological correlates of resilience and personality traits within the triple resting-state brain networks assessed by 7-Tesla ultra-high field MRI
Source: Sci Rep. 2021 Jun 2;11:11564. doi: 10.1038/s41598-021-91056-y (PMC8172832; doi:10.1038/s41598-021-91056-y)
Supplement: Supplementary file 1 — Supplementary Table 1. [file 41598_2021_91056_MOESM1_ESM.docx]

**Supplementary Table 1:**

Individual values achieved on the resilience Scale (RS-25) and the single scales of the NEO-FFI.

Sex: 0 = female, 1 = male

| **Nr.** | **Sex** | **Age** | **RS_25** | **NEO:**  **Neuroticism** | **NEO:**  **Extraversion** | **NEO:**  **Openness** | **NEO:**  **Agreeable-**  **ness** | **NEO:**  **Conscien-**  **tiousness** |
| --- | --- | --- | --- | --- | --- | --- | --- | --- |
| 1 | 0 | 35 | 154 | 11 | 27 | 15 | 34 | 35 |
| 2 | 0 | 21 | 138 | 14 | 29 | 30 | 35 | 31 |
| 3 | 1 | 22 | 122 | 18 | 13 | 27 | 36 | 36 |
| 4 | 1 | 49 | 142 | 11 | 29 | 27 | 30 | 41 |
| 5 | 1 | 22 | 130 | 16 | 27 | 33 | 35 | 30 |
| 6 | 0 | 36 | 171 | 7 | 42 | 40 | 39 | 47 |
| 7 | 1 | 41 | 159 | 15 | 34 | 30 | 35 | 38 |
| 8 | 1 | 28 | 136 | 13 | 26 | 36 | 33 | 33 |
| 9 | 0 | 19 | 140 | 24 | 21 | 31 | 30 | 25 |
| 10 | 0 | 46 | 147 | 26 | 21 | 31 | 34 | 42 |
| 11 | 0 | 27 | 155 | 9 | 39 | 23 | 43 | 42 |
| 12 | 1 | 26 | 149 | 16 | 31 | 32 | 32 | 38 |
| 13 | 1 | 31 | 130 | 30 | 23 | 23 | 32 | 31 |
| 14 | 1 | 29 | 149 | 13 | 35 | 33 | 37 | 40 |
| 15 | 1 | 21 | 134 | 8 | 32 | 35 | 35 | 30 |
| 16 | 0 | 19 | 152 | 11 | 32 | 34 | 44 | 39 |
| 17 | 1 | 26 | 154 | 8 | 30 | 29 | 28 | 29 |
| 18 | 0 | 25 | 148 | 14 | 26 | 33 | 35 | 34 |
| 19 | 0 | 21 | 134 | 25 | 21 | 30 | 30 | 19 |
| 20 | 1 | 25 | 156 | 10 | 39 | 42 | 25 | 35 |
| 21 | 0 | 22 | 162 | 5 | 35 | 26 | 40 | 42 |
| 22 | 0 | 24 | 150 | 7 | 32 | 31 | 33 | 36 |
| 23 | 0 | 22 | 120 | 19 | 25 | 30 | 37 | 40 |
| 24 | 1 | 51 | 165 | 9 | 27 | 28 | 37 | 39 |
| 25 | 0 | 23 | 150 | 12 | 32 | 35 | 34 | 39 |
| 26 | 0 | 48 | 170 | 9 | 36 | 34 | 35 | 45 |
| 27 | 1 | 34 | 136 | 16 | 26 | 30 | 31 | 37 |
| 28 | 1 | 29 | 123 | 13 | 35 | 32 | 37 | 29 |
| 29 | 0 | 23 | 165 | 5 | 34 | 32 | 44 | 48 |
| 30 | 0 | 31 | 152 | 18 | 33 | 29 | 35 | 37 |
| 31 | 1 | 25 | 129 | 31 | 21 | 40 | 18 | 24 |
| 32 | 1 | 22 | 121 | 21 | 20 | 23 | 37 | 22 |
