# Supplementary material for: Common neurobiological correlates of resilience and personality traits within the triple resting-state brain networks assessed by 7-Tesla ultra-high field MRI
Source: Sci Rep. 2021 Jun 2;11:11564. doi: 10.1038/s41598-021-91056-y (PMC8172832; doi:10.1038/s41598-021-91056-y)
Supplement: Supplementary file 3 — Supplementary Table 3. [file 41598_2021_91056_MOESM3_ESM.docx]

**Supplementary Table 3:**

Additional correlation analysis performed between the personality traits extraversion and conscientiousness and the ReHo values obtained from the subregions of the salience network (SN).

The correlations were performed with the same procedure as described for the network-level analysis in the methods section. Statistically significant results (after performing a permutation test for multiple comparisons in the Pearson correlation analyses) are marked with asterixis.

Regarding conscientiousness, this additional analysis revealed a significant negative association with ReHo (r = - 0.35, p = 0.05) in the subregion composed of anterior cingulate cortex, medial prefrontal cortex, supplementary motor area. Further, there was a significant positive correlation with the ReHo value (r = 0.46, p = 0.01) in cerebellar lobule VI.

Regarding extraversion and the parameter ReHo for which we observed a negative association in the whole SN, a significant negative association (r = - 0.37, p = 0.03) was observed in the subregion composed of anterior cingulate cortex, medial prefrontal cortex, supplementary motor area as well as with the left precuneus (r = - 0.49, p = 0.01). Further, extraversion correlated significantly positive with ReHo in the left thalamus (r = 0.39, p = 0.03) and in the cerebellar lobule VI (r = 0.47, p = 0.01).

| **Regions** | **NEO-FFI Extraversion** | | **NEO-FFI**  **Conscientiousness** | |
| --- | --- | --- | --- | --- |
|  | **r** | ***p*** | **r** | ***p*** |
| *Left Middle Frontal Gyrus* | 0.00 | 0.99 | -0.09 | 0.65 |
| *Left Insula* | 0.00 | 0.97 | -0.17 | 0.36 |
| *Anterior Cingulate Cortex, Medial Prefrontal Cortex, Supplementary Motor Area* | **-0.37** | **0.03*** | **-0.35** | **0.05*** |
| *Right Middle Frontal Gyrus* | -0.11 | 0.55 | -0.04 | 0.83 |
| *Right Insula* | -0.19 | 0.31 | -0.16 | 0.38 |
| *Left Lobule VI, Crus I* | 0.19 | 0.28 | 0.10 | 0.55 |
| *Right Lobule VI, Crus I* | -0.05 | 0.78 | -0.15 | 0.43 |
| *Anterior Salience Network Combined* | -0.32 | 0.07 | -0.34 | 0.05 |
| *Left Middle Frontal Gyrus* | -0.17 | 0.34 | -0.13 | 0.50 |
| *Left Supramarginal Gyrus, Inferior Parietal Gyrus* | -0.18 | 0.33 | -0.22 | 0.22 |
| *Left Precuneus* | **-0.49** | **0.01*** | -0.30 | 0.09 |
| *Right Midcingulate Cortex* | -0.06 | 0.71 | 0.10 | 0.59 |
| *Right superiorparietal gyrus, Precuneus* | -0.08 | 0.68 | 0.00 | 1.00 |
| *Right Supramarginal Gyrus, Inferior Parietal Gyrus* | -0.31 | 0.09 | -0.28 | 0.13 |
| *Left Thalamus* | **0.39** | **0.03*** | 0.21 | 0.25 |
| *Lobule VI* | **0.47** | **0.01*** | **0.46** | **0.01*** |
| *Left Posterior Insula, Putamen* | -0.19 | 0.29 | 0.15 | 0.40 |
| *Right Thalamus* | 0.03 | 0.86 | -0.03 | 0.85 |
| *Lobule VI* | -0.07 | 0.68 | 0.15 | 0.41 |
| *Right Posterior Insula* | 0.24 | 0.19 | 0.21 | 0.25 |
| *Posterior Salience Network Combined* | -0.30 | 0.09 | -0.24 | 0.18 |
